# Clinicopathological Analysis of Ovarian Neoplasms at a Tertiary Care Teaching Institute of North Malabar: A Four-Year Retrospective Study

**DOI:** 10.7759/cureus.77574

**Published:** 2025-01-17

**Authors:** Janaky Ramakrishnan, Mary Nandini Singh, Benzy Paul, Sathi Puthen Parambath

**Affiliations:** 1 Pathology, Kunhitharuvai Memorial Charitable Trust (KMCT) Medical College, Kozhikode, IND

**Keywords:** borderline tumours, ovarian neoplasms, serous carcinoma, sex cord, teratoma

## Abstract

Background

Ovarian neoplasms represent a significant risk to women's health worldwide. Approximately 90% of ovarian neoplasms are of epithelial origin, representing the predominant category and comprising many subtypes, such as mucinous and serous. The incidence of ovarian cancer increases with age. Serous carcinoma, the predominant ovarian cancer observed in older individuals, is associated with a poor prognosis. A precise histopathological diagnosis is essential for successful management and therapy planning, as the histological subtype influences treatment modalities and prognostic outcomes.

Aim

This study aimed to determine the clinical symptomatology, age distribution, tumor laterality, gross appearance, and histopathological spectrum of ovarian tumors.

Materials and methods

This retrospective study was conducted at a tertiary care center in the Malabar region of North Kerala. All ovarian neoplasms received in the department of pathology as part of the diagnostic work-up from September 2019 to September 2023 were taken for analysis. Gross and microscopic features of resection specimens, along with relevant clinical data, were reviewed and analyzed from previous records. The ovarian neoplasms were categorized according to the latest WHO classification.

Results

The total sample size for ovarian neoplasms was 455. The age range was from 11 to 80 years. The predominant presenting complaint was abdominal pain (28%, n=128), followed by pelvic discomfort (16%, n=75). Surface epithelial ovarian tumors represented the predominant histological subtype with 329 (72%) cases, while the germ cell tumor category comprised 84 (19%) cases, followed by sex cord-stromal tumors with 24 (5%), mixed ovarian tumors with 11 (2%), and miscellaneous tumors with 7 (2%) cases. The majority of tumors in the surface epithelial (n=329) category were benign cystadenomas/cystadenofibromas, with 291 (88.4%) cases, while borderline and malignant epithelial tumors constituted 9 (2.8%) and 29 (8.8%) cases, respectively. The most common benign ovarian neoplasm was serous cystadenoma, with 208 (45.7%) cases, followed by mature cystic teratoma with 80 (17.6%) cases. The most common malignant tumor was serous carcinoma, with 15 (3.3%) cases. Metastasis of the ovary was seen in four cases, one of which was a bilateral ovarian metastasis from a uterine endometrial stromal sarcoma. We also encountered a rare case of small cell carcinoma of the ovary in a 26-year-old woman.

Conclusion

Ovarian tumors encompass a wide spectrum of neoplasms ranging from benign to highly malignant lesions, presenting significant diagnostic and management challenges. While benign tumors like serous cystadenomas often cause minimal morbidity and are readily treated surgically, malignant ovarian tumors, including high-grade serous carcinoma, are associated with a grave prognosis due to their often delayed diagnosis and aggressive nature. Early detection remains paramount to reducing the high morbidity and mortality associated with ovarian malignancies.

## Introduction

Ovarian tumors are a growing global health concern, contributing to increasing morbidity and mortality among women. Ovarian cancer is the eighth most prevalent malignancy among women worldwide, with more than 324,603 new cases reported globally in 2022 [1]. In India, ovarian cancer ranks as the third most prevalent cancer among women, following cervical and breast cancers. In developing countries like India, the majority of ovarian malignancies are diagnosed at advanced stages (III-IV), significantly impacting survival rates, which are around 45% over five years [2,3]. Ovarian neoplasms occur across all age groups but demonstrate distinct patterns based on age. In women of reproductive age, the majority of ovarian cysts are non-neoplastic, primarily consisting of functional cysts, tubo-ovarian endometriotic cysts, and corpus luteal cysts. The benign cystic neoplasms in the reproductive age group are mostly serous cystadenomas or dermoid cysts, which do not pose a serious problem and can be surgically excised. Conversely, ovarian malignancies are more common in older women, especially after menopause. Clinical presentations can range from asymptomatic cases to pelvic pain and menstrual irregularities. Some women may experience nonspecific symptoms like vague abdominal discomfort and urinary disturbances. Ovarian neoplasms are influenced by several risk factors, including genetic mutations such as BRCA1/BRCA2 and Lynch syndrome, a family history of ovarian or related cancers, and reproductive factors such as nulliparity, early menarche, and late menopause, which increase lifetime ovulations. Lifestyle factors, including obesity and smoking, also contribute, with smoking particularly linked to mucinous tumors. Hormone replacement therapy (HRT) can elevate the risk for ovarian tumors, while protective factors like oral contraceptive use, breastfeeding, and tubal ligation can reduce it. The anatomic location of the ovary presents unique challenges for gynecologists because, unlike cervical cancers with established screening protocols, ovarian malignancies often go undetected, and many patients have advanced disease at presentation [4, 5]. For women with hereditary risk for ovarian cancer (e.g., BRCA mutations), prophylactic risk-reducing salpingo-oophorectomy has emerged as a critical preventive strategy. Histopathology is fundamental to accurately categorizing ovarian neoplasms. A histopathologic diagnosis based on cytological and architectural features, ancillary studies like immunohistochemistry, and molecular testing when appropriate, along with clinical staging, will help to determine appropriate management strategies and prognostic outcomes. The purpose of this study was to determine the frequency of ovarian tumors in the north Malabar region of Kerala. The other variables studied were the mode of clinical presentation, age distribution, parity, laterality of ovarian tumor, gross appearance, and histological subtypes based on the WHO classification of ovarian neoplasms [6].

## Materials and methods

Source of data

A four-year, record-based retrospective study of the histopathological spectrum of 455 ovarian neoplasms was conducted in the Department of Pathology at Kunhitharuvai Memorial Charitable Trust (KMCT) Medical College, Manassery, Mukkam, Kozhikode District, Kerala, from September 2019 to August 2023, with approval from the KMCT Medical College Institutional Ethics Committee (IEC Ref No: IECKMCT/29/2024-26.02.2024).

Inclusion criteria

We included all ovarian neoplasms, both benign and malignant, that were submitted for histopathology analysis, regardless of their clinical data or stage of the disease. Ovarian tumors found incidentally in routine hysterectomy specimens were also included in the study.

Exclusion criteria

Non-neoplastic ovarian lesions such as follicular cysts, simple serous cysts of the ovary, tubo-ovarian masses (endometriotic cysts), and polycystic ovaries (PCOD) were excluded from the study.

Methods of data collection

The final sample size for ovarian neoplasms was 455. The presenting clinical complaint, duration of symptoms, parity, laterality, family history, and relevant laboratory investigations were obtained from the patients' histopathology requisition forms. Gross size and appearance of the ovarian tumors (purely cystic, purely solid, cystic-solid) were noted from the pathology request forms. Histopathology slides were retrieved and reanalyzed, and necessary data were entered into an MS Excel spreadsheet. Paraffin blocks were retrieved whenever needed for additional studies. Ovarian tumors were classified according to the WHO 2022 classification of ovarian tumors [6]. Statistical analysis was performed after entering the data into an MS Excel spreadsheet for the generation of tables, frequencies, and percentage calculations.

## Results

A total of 646 ovarian lesions were collected; 191 were non-neoplastic and excluded from the study. The remaining 455 cases were analyzed based on clinical presentation, age, parity, laterality, gross size, consistency, and histological characteristics. This study categorized ovarian tumors into four age categories, as illustrated in Table 1. The first age group, encompassing 0-18 years (from infancy to adolescence), comprised 15 (3.4%) cases. This was succeeded by the 19-40 years age group (reproductive age), which accounted for 174 (38.2%) cases. The perimenopausal age group (41-50 years) recorded 122 (26.8%) cases, while the postmenopausal age group (≥ 51 years) had 144 (31.6%) cases (Table 2). The observed age range was from 11 to 80 years. The youngest, an 11-year-old prepubertal girl, presented with a benign mucinous cystadenoma, while the oldest, an 80-year-old woman, was diagnosed with ovarian endometrioid adenocarcinoma. The most common clinical presentation was abdominal pain, observed in 128 (28%) cases, whereas pelvic discomfort was the primary complaint in 75 (16%) cases, as detailed in Table 3. Fifty-eight percent of patients had one or more symptoms at clinical presentation.

**Table 1:** Age-wise distribution of ovarian neoplasms based on tumor category.

Age range (years)	Epithelial tumours	Germ cell tumours	Sex cord stromal tumours	Mixed ovarian tumours (Epithelial + Germ cell/ Sex cord stromal)	Miscellaneous	Total no. of cases
≤18 Years	9 (60%)	5 (33.3%)	1 (6.7%)	0 (0%)	0 (0%)	15
19-40 Years	110 (63.3%)	54 (31%)	2 (1 %)	5 (3%)	3 (1.7%)	174
41-50 Years	95 (78%)	16 (13%)	8 (6.6%)	1 (0.8%)	2 (1.6%)	122
≥51 Years	115 (80%)	9 (6.2%)	13 (9%)	5 (3.4%)	2 (1.4%)	144
Total no. of cases	329	84	24	11	7	455

**Table 2:** Age-wise distribution based on the nature of ovarian tumors.

Age range (years)	Benign	Borderline	Malignant	Total no. of cases (%)
≤18 Years	13 (86.6%)	1 (6.7%)	1 (6.7%)	15 (100.0%)
19-40 Years	159 (91.4%)	4 (2.3%)	11 (6.3 %)	174 (100.0%)
41-50 Years	110 (90.2%)	3 (2.5%)	9 (7.3%)	122 (100.0%)
≥51 Years	118 (82%)	2 (1.3%)	24 (16.7%)	144 (100.0%)
Total no. of cases	400	10	45	455 (100.0%)

**Table 3:** Clinical presentation of ovarian neoplasms (n=455). 58% of patients had one or more symptoms at presentation.

Main presenting complaint	n (%)
Abdominal pain	128 (28%)
Pelvic discomfort	75 (16%)
Acute pain/torsion	9 (2%)
Abdominal distension	45 (10%)
Mass per abdomen	40 (9%)
Heavy menstrual bleeding (HMB)	27 (6%)
Irregular menstrual cycles	18 (4%)
Post menopausal bleeding	18 (4%)
Urinary complaints	45 (10%)
Constipation	13 (3%)
Mass descending per vaginum (PV)	10 (2%)
Asymptomatic	14 (3%)
Pregnancy complicated by ovarian cyst	13 (3%)

Table 4 presents the tabulated distribution of parity, menopausal state, laterality, gross size, and consistency. The parous group exhibited the highest frequency of ovarian tumors, with 388 (85%) cases. Among the 45 malignant cases seen, over half (53.3%) were diagnosed in postmenopausal women. Left-sided ovarian tumors, accounting for 52% of cases, were more prevalent than right-sided neoplasms, which constituted 38%. Among the 455 cases, 400 ovarian tumors (88%) were classified as benign in nature, 10 cases (2%) as borderline tumors, and 45 cases (10%) as ovarian malignancies. Bilateral ovarian tumors were observed in 46 (10%) cases. The size distribution of cystic-to-solid ovarian tumors has a wide range. The smallest ovarian tumor measured 1.5 cm in a 39-year-old woman with bilateral ovarian metastases, while the largest tumor measured 30 cm in a 54-year-old woman with mucinous carcinoma. The predominant size group for ovarian neoplasms was ≤ 10 cm (61%), followed by 11-20 cm (35%). The macroscopic characteristics of ovarian tumors were also documented. Cystic morphology was observed in 81%, solid-cystic morphology in 14%, and entirely solid ovarian tumors in 5% of cases.

**Table 4:** Distribution of parity, menopausal status, laterality, and gross size and appearance based on the nature of ovarian tumors.

Sl No.	Variables studied	Nature of ovarian tumours			Total no. of cases (%)
1	Parity	Benign	Borderline	Malignant	
	Unmarried and nulliparous	36	1	3	40 (9.0%)
	Married and nulliparous	22	0	5	27 (6.0%)
	Parous	342	9	37	388 (85.0%)
	Total no. of cases	400	10	45	455 (100.0%)
2	Menopausal status	Benign	Borderline	Malignant	n
	Premenopausal	282	8	21	311 (68.4%)
	Postmenopausal	118	2	24	144 (31.6%)
	Total no. of cases	400	10	45	455 (100.0%)
3	Laterality of tumors	Benign	Borderline	Malignant	n
	Right	155	1	17	173 (38.0%)
	Left	204	8	24	236 (52.0%)
	Bilateral	41	1	4	46 (10.0%)
	Total no. of cases	400	10	45	455 (100.0%)
4	Gross size of tumour (in cms)	Benign	Borderline	Malignant	n
	≤10 cms	249	4	25	278 (61.0%)
	11-20 cms	139	5	13	157 (35.0%)
	21-30 cms	12	1	7	20 (4.0%)
	Total no. of cases	400	10	45	455 (100.0%)
5.	Consistency of tumour	Benign	Borderline	Malignant	n
	Cystic	361	7	1	369 (81.0%)
	Cystic and solid (mixed)	21	3	39	63 (14.0%)
	Solid	18	0	5	23 (5.0%)
	Total no. of cases	400	10	45	455 (100.0%)

**Figure 1:**
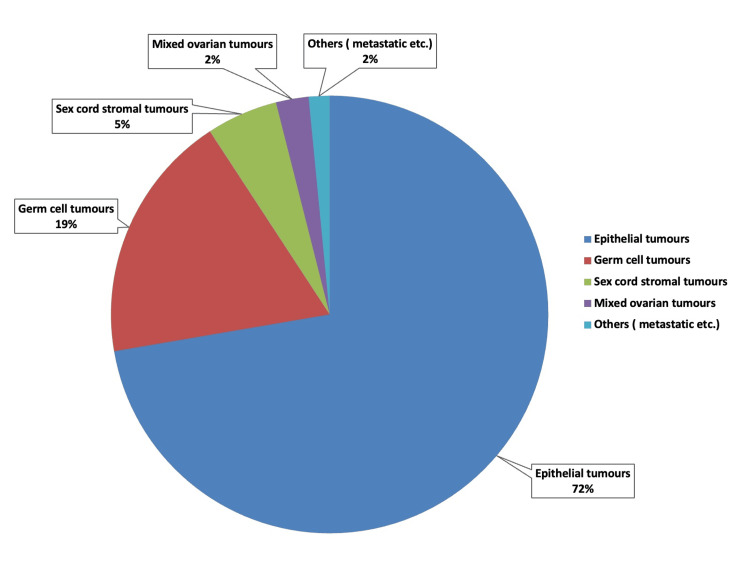
Distribution of ovarian tumors.

**Figure 2:**
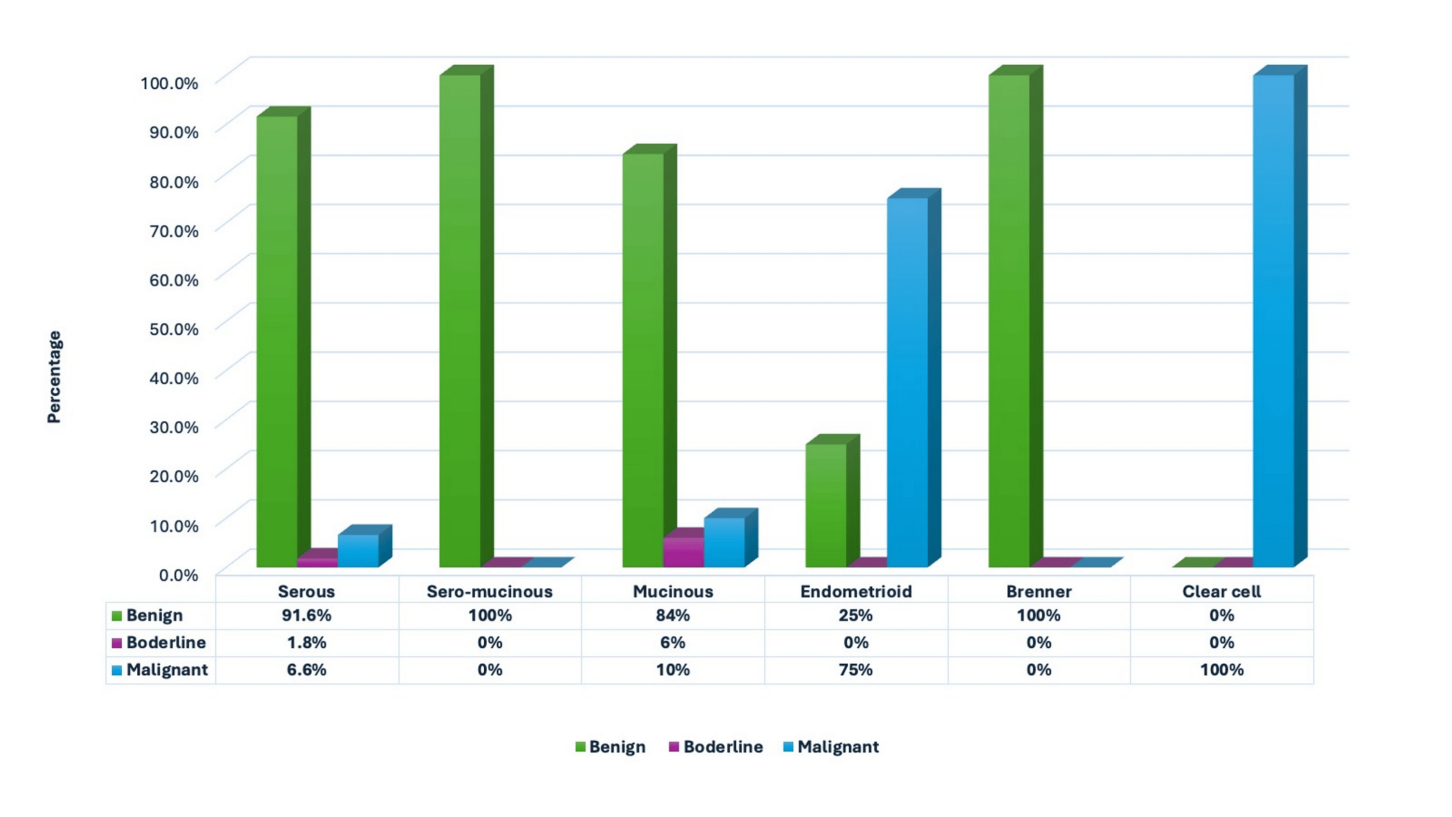
Distribution of surface epithelial ovarian neoplasms based on the nature of the lesion.

The distribution of ovarian tumors is illustrated in Table 5 and Figure 1. Figure 2 depicts the distribution of ovarian tumors based on the nature of the lesion. Seventy-two percent of ovarian tumors were of surface epithelial origin. The next prevalent type seen was germ cell tumors (19%), followed by sex cord stromal tumors (5%). The fourth category, mixed ovarian cancers (i.e., epithelial tumors coexisting with either germ cell tumors or sex cord stromal tumors), included 11 (2%) cases. The miscellaneous category, encompassing metastatic carcinomas, had 7 (2%) cases.

**Table 5:** Distribution of ovarian neoplasms.

Category of ovarian neoplasms	Histopathological subtypes in each category	Nature of tumour	No. of cases (n)	Percentage
Epithelial Tumours	Serous	Benign	208	45.7
		Borderline	4	0.9
		Malignant	15	3.3
	Sero-mucinous	Benign	5	1.1
		Borderline	0	0.0
		Malignant	0	0.0
	Mucinous	Benign	76	16.7
		Borderline	5	1.1
		Malignant	9	2.0
	Endometrioid	Benign	1	0.2
		Borderline	0	0.0
		Malignant	3	0.7
	Transitional cell tumor (Brenner tumor)	Benign	1	0.2
		Borderline	0	0.0
		Malignant	0	0.0
	Clear cell tumours	Benign	0	0.0
		Borderline	0	0.0
		Malignant	2	0.4
Sex cord-stromal tumors	Fibro-thecomas	Benign	15	3.3
	Leydig cell tumour		1	0.2
	Granulosa-cell tumor	Malignant	6	1.3
	Sertoli-Leydig cell tumor		2	0.4
Germ cell tumors	Mature cystic teratoma	Benign	80	17.6
	Monodermal teratoma		2	0.4
	Immature teratoma	Malignant	1	0.2
	Dysgerminoma		1	0.2
Mixed ovarian tumours	Mucinous cystadenoma + Brenner Tumour	Benign	1	0.2
	Serous cystadenoma + Brenner Tumour		2	0.4
	Mucinous cystadenoma + Teratoma		4	0.9
	Serous cystadenoma + Teratoma		3	0.7
	Mucinous Carcinoma + Teratoma	Malignant	1	0.2
Miscellaneous tumours	Ovarian Leiomyoma	Benign	1	0.2
	Atypical Endometriotic Cyst, Ovary	Borderline	1	0.2
	Small cell carcinoma, hypercalcemic type	Malignant	1	0.2
	Metastatic (non-ovarian) tumours		4	0.9
Total no. of cases			455	100.0%

**Figure 3:**
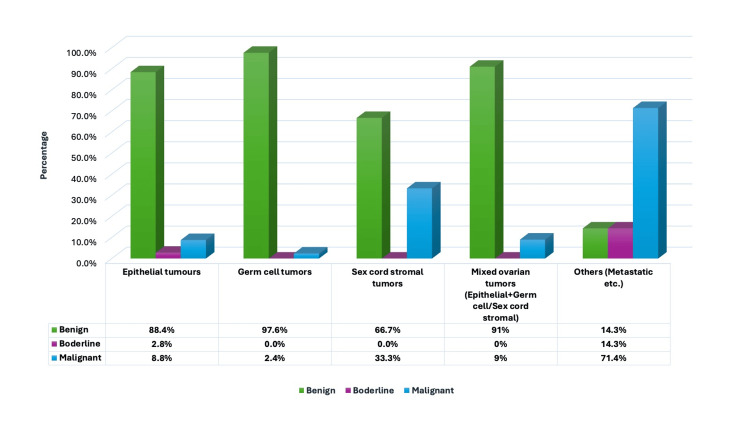
Distribution of ovarian tumors based on the nature of the lesion.

**Figure 4:**
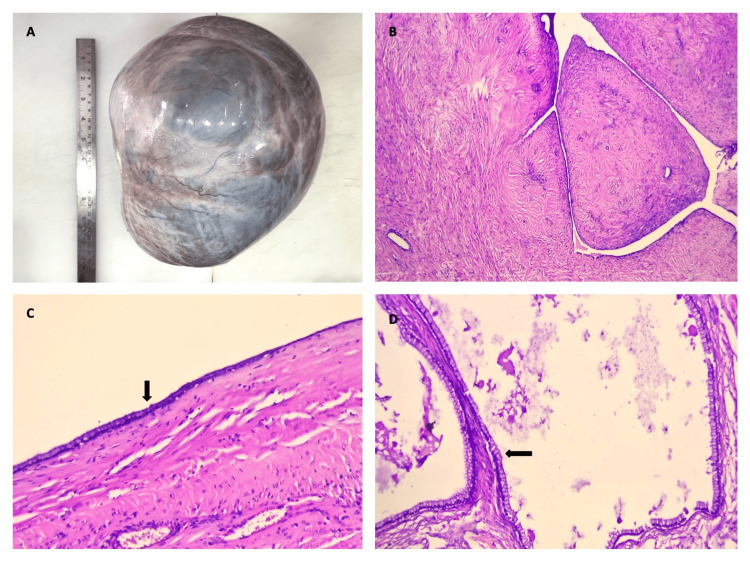
4A: Gross specimen of serous cystadenoma, ovary; 4B: Photomicrograph of serous cystadenofibroma, ovary (H&E, 4x); 4C: Photomicrograph of sero-mucinous cystadenoma, ovary (H&E, 10x); 4D: Photomicrograph of mucinous cystadenoma, ovary (H&E, 40x).

**Figure 5:**
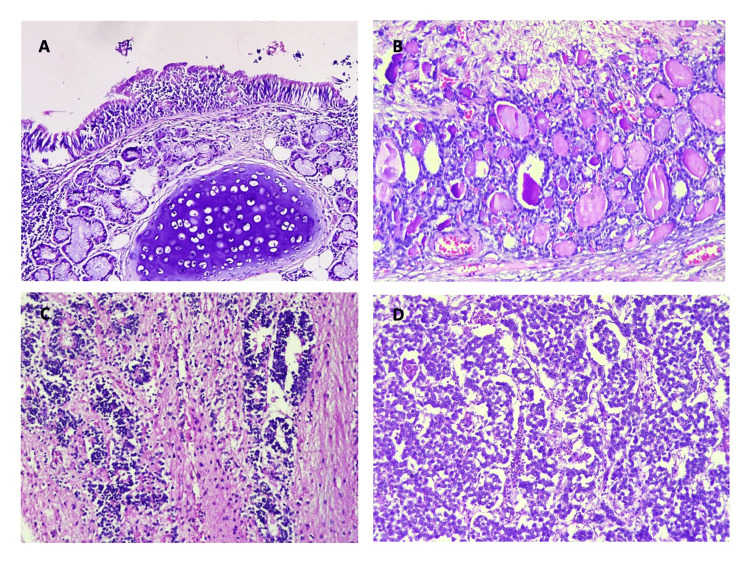
5A: Photomicrograph of mature teratoma, ovary. Respiratory epithelium and mature cartilage is seen (H&E, 10x); 5B: Photomicrograph of monodermal teratoma (struma ovarii) (H&E, 10x); 5C: Photomicrograph of immature teratoma, ovary with primitive neuroepithelium (H&E, 10x); 5D: Photomicrograph of dysgerminoma, ovary (H&E, 10x).

**Figure 6:**
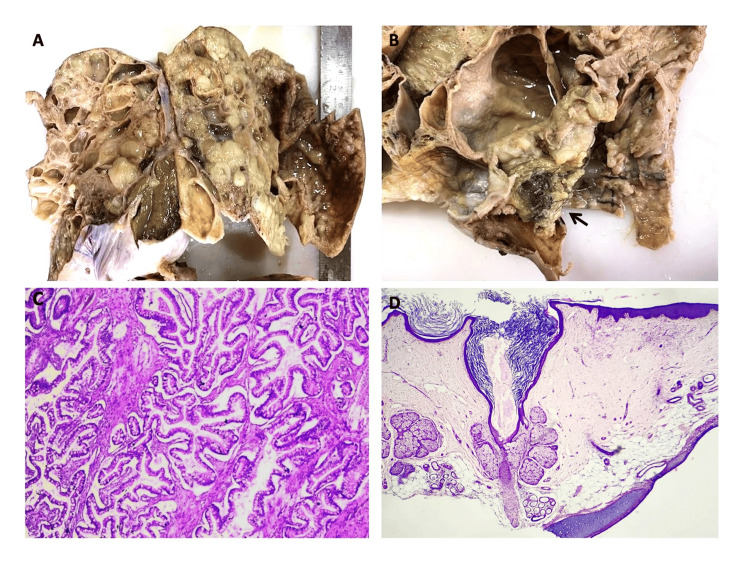
6A and 6B: Gross specimen of mucinous carcinoma with mature cystic teratoma, ovary; in 6B, hair tufts are seen in the complex cystic mass (black arrow); 6C: Photomicrograph of mucinous carcinoma component (expansile pattern) (H&E, 4x); 6D: Photomicrograph of teratoma component with skin and mature cartilage (H&E, 4x).

**Figure 7:**
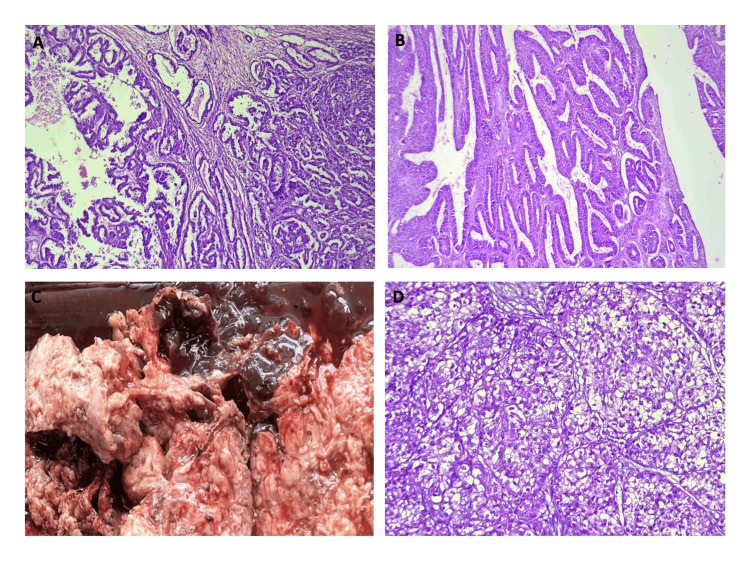
7A: Photomicrograph of serous carcinoma, ovary (H&E, 10x); 7B: Photomicrograph of endometrioid adenocarcinoma, ovary (H&E, 10x); 7C: Gross specimen of clear cell carcinoma, ovary; 7D: Photomicrograph of clear cell carcinoma, ovary (H&E, 10x).

**Figure 8:**
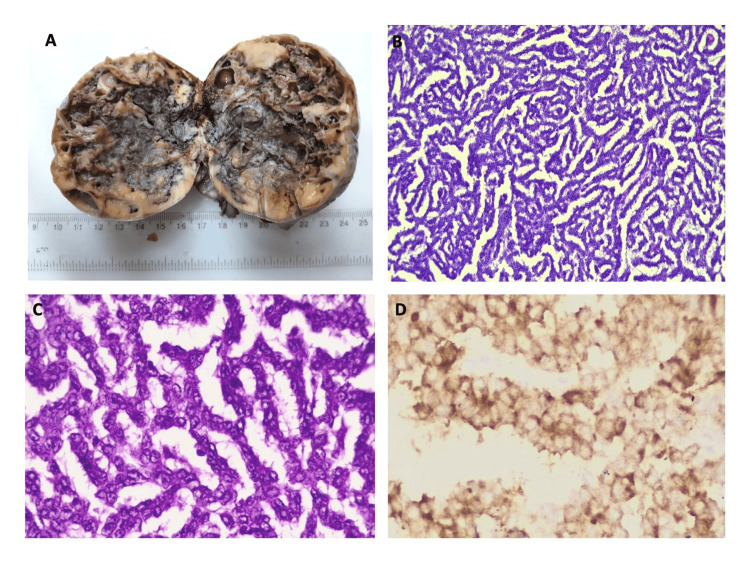
8A: Gross specimen of adult granulosa cell tumor, ovary; 8B: Photomicrograph of tumor cells arranged in a gyriform pattern (H&E, 10x); 8C: Photomicrograph showing small polygonal cells with pale, angulated, and grooved nuclei (H&E, 40x); 8D: IHC photomicrograph (Inhibin) showing positivity in tumor cells (40x). IHC: Immunohistochemistry.

**Figure 9:**
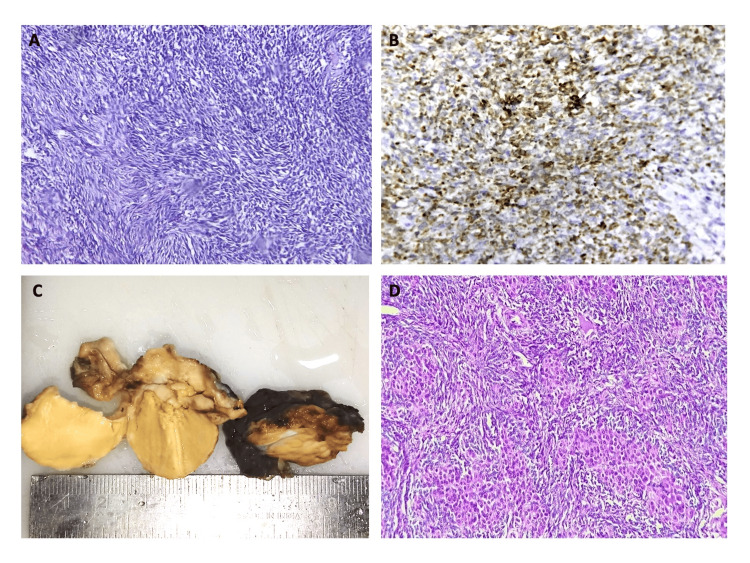
9A: Photomicrograph of ovarian fibro-thecoma (H&E, 40x); 9B: IHC photomicrograph (Inhibin) showing focal positivity in tumor cells (40x); 9C: Gross specimen of Sertoli Leydig cell tumor, ovary; 9D: Photomicrograph of Sertoli Leydig cell tumor, ovary (H&E, 10x). IHC: Immunohistochemistry.

**Figure 10:**
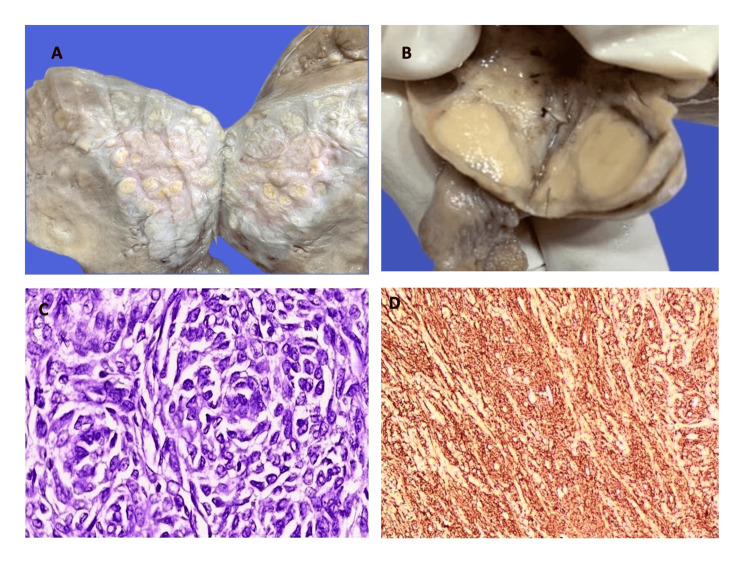
10A: Gross specimen of uterus (myometrium) involved by ESS; 10B: Gross specimen of ipsilateral ovary with metastatic ESS tumor deposit; 10C: Photomicrograph of ovary showing metastatic tumor deposit of ESS (H&E, 40x); 10D: IHC photomicrograph (CD10) showing diffuse positivity in metastatic ovarian tumor cells (10x). ESS: Endometrial stromal sarcoma; IHC: Immunohistochemistry.

Figure 3 depicts the distribution of surface epithelial ovarian neoplasms according to the nature of the lesion. The serous group had the maximum number of tumors (n = 227). Serous cystadenoma, illustrated in Figures 4A and 4B, was the predominant tumor, including 208 (91.6%) cases. All five seromucinous tumors were classified as benign cystadenomas, as illustrated in Figure 4C. In the group of mucinous tumors, mucinous cystadenoma, which made up 76 (84% of the cases) shown in Figure 4D, was the most common type. In the germ cell tumor group, mature cystic teratoma, shown in Figure 5A, was the most common benign tumor, making up 97.6% of the cases. Monodermal teratoma (struma ovarii) was also observed, as illustrated in Figure 5B. We documented one case of immature teratoma with primitive neuroepithelium, illustrated in Figure 5C, and one instance of dysgerminoma, depicted in Figure 5D. We had one case of mucinous carcinoma arising in a mature cystic teratoma of the ovary, as shown in Figures 6A-6D. Among the ten cases classified as borderline tumors, four were of serous origin, and five were of mucinous origin. Table 6 illustrates the distribution of malignant ovarian tumors. The predominant malignancy observed was serous carcinoma, illustrated in Figure 7A, with 15 (33.3%) instances, followed by mucinous carcinoma with 10 (22.2%) cases. Additional malignant epithelial tumors observed include endometrioid adenocarcinomas (Figures 7B) and clear cell carcinomas (Figures 7C-7D). We had six cases of granulosa cell tumors (one juvenile and five adult types), as illustrated in Figures 8A-8D (1.3%). Within the sex-cord stromal category, most tumors were benign and classified as fibrothecomas, as illustrated in Figures 9A-9B, which comprise 15 (3.3%) cases. We had two cases of Sertoli Leydig cell tumors, as depicted in Figures 9C and 9D. Within the miscellaneous group, we identified four instances of ovarian metastasis, one of which originated from a uterine endometrial stromal sarcoma, as illustrated in Figures 10A to 10D. 

**Table 6:** Distribution of malignant ovarian tumors.

Malignant tumours	No. of cases	Percentage
Serous carcinoma	15	33.3
Mucinous carcinoma	10	22.2
Endometriod carcinoma	3	6.7
Clear cell carcinoma	2	4.4
Granulosa cell tumor	6	13.4
Sertoli Leydig cell tumour	2	4.4
Immature Teratoma	1	2.2
Dysgerminoma	1	2.2
Small cell carcinoma, hypercalcaemic type	1	2.2
Metastasis to ovary	4	9.0
Total no. of cases	45	100.0%

## Discussion

Ovarian neoplasms are traditionally divided into four groups: epithelial, sex cord-stromal, germ cell, and miscellaneous tumors. Epithelial tumors constitute two-thirds of all ovarian tumors, and their malignant counterparts represent 80% of all ovarian malignancies, with serous carcinomas being the most common. A newer model divides surface epithelial tumors into two groups based on origin, tumorigenic pathways, and biological behavior. Type I epithelial tumors are low-grade and have a relatively indolent clinical course, while Type II tumors are clinically aggressive high-grade neoplasms. The majority of Type II tumors are high-grade serous carcinomas (HGSCs), with 80% occurring in FIGO stages III and IV and often associated with poor prognosis. Immunohistochemistry and molecular testing are increasingly used, but histomorphology remains the mainstay in categorizing ovarian tumors. Recent developments in molecular profiling have shown that more than 90% of adult granulosa cell tumors harbor FOXL2 mutations, and more than 90% of Sertoli-Leydig cell tumors have DICER1 mutations [6].

In our study of 455 ovarian tumors, the overall age range was 11 to 80 years, similar to studies conducted by Garg N et al. [7], Mankar DV et al. [8], and Modepalli N et al. [9]. The highest incidence was found in the reproductive age group with 174 (38.2%) cases, a finding also observed by Pilli GS et al. [10]. The overall mean and median ages were 43.2 years and 44 years, respectively. The mean ages for the four ovarian tumor categories (surface epithelial, germ cell, sex cord-stromal, and metastatic tumors) were 44.7 years, 36.1 years, 50.8 years, and 48.8 years, respectively. The mean age of germ cell tumors in our study is consistent with the findings of Kos M et al. at 34.7 years [11]. Age is considered an independent prognostic factor for ovarian malignancies. Research indicates that the mean age varies significantly among tumor subgroups, with a median age at diagnosis of approximately 63 years globally. However, in the Indian subpopulation, the median age is frequently reported as less than 55 years. The median age of 53 years and the mean age of 49.3 years for ovarian malignancies in our study are comparable to those reported by Gangane NM et al. [12], who reported a median age of 50 years and a mean age of 49.06 years. These findings underscore a notable demographic disparity in the manifestation of ovarian cancer among Indian women compared to their Western counterparts, highlighting the necessity for customized awareness and screening initiatives for younger populations in India.

In the current investigation, the majority of tumors were unilateral, with 409 cases (90%). We also discovered that left-sided tumors were more prevalent than right-sided tumors. Abdominal discomfort was the prevalent clinical manifestation observed in 128 (28%) cases, consistent with the study conducted by Anitha DP et al. [13]. Among the 455 ovarian tumors, 400 were benign (88%), 45 were malignant (10%), and 10 were borderline tumors (2%), findings consistent with those of Thakkar NN et al. [14], as illustrated in Table 7.

**Table 7:** Comparison study of frequencies of benign, borderline, and malignant ovarian tumors.

Categories	Present study	Anitha Das et al. (2024) [13]	Thakkar N et al. (2015) [14]	Gupta N et al. (2007) [15]
Benign	n=400 (88%)	n=115 (82%)	n=109 (84.6%)	n=135 (63.7%)
Borderline	n=10 (2%)	n=5 (3.5%)	n=3 (2.3%)	n=11 (5.2%)
Malignant	n=45 (10%)	n=20 (14.5%)	n=17 (12.6%)	n=66 (31.3%)
Total no. of cases	455	140	129	212

Figure 1 illustrates the categorization of ovarian cancers into five groups: epithelial, germ cell, sex cord-stromal, mixed ovarian, and miscellaneous tumors. The results were analogous to those of the study by Gupta N et al. [15]. Upon examining the age-specific distribution of ovarian tumors, we observed that epithelial tumors had the highest prevalence across all age categories, as illustrated in Table 1. Germ cell tumors were the second most prevalent neoplasm observed in adolescents and individuals in reproductive and perimenopausal age groups. In the postmenopausal cohort, the second most prevalent neoplasm observed was sex cord stromal tumors. These findings closely resemble the study conducted by Gautam KB et al. [16].

The most prevalent epithelial tumor was serous cystadenoma, with 208 (91.6%) cases. The most prevalent mucinous tumor was mucinous cystadenoma, with 76 (84%) cases. Of the 29 malignant epithelial tumors, 15 (51.7%) were serous carcinomas. Mature cystic teratomas dominated the germ cell category, accounting for 80 (97.6%) cases. The most prevalent benign sex cord stromal tumor was fibrothecomas, with 15 (62.5%) instances. Granulosa cell tumor was the most frequent malignancy in the sex cord stromal tumor category, with 6 (25%) cases. Bukhari U et al. [17] found similar results in their literature review.

In our study, we had 15 cases of ovarian cysts detected during pregnancy (3.2%). Literature shows that ovarian cysts are relatively common during pregnancy, with reported incidences ranging from 1% to 5.3% [18]. Nine of the 15 cases seen during pregnancy were benign serous cystadenomas (60%), followed by four cases of mucinous cystadenomas (26.7%) and one case of an adult cystic teratoma (6.6%). A 27-year-old pregnant woman with abdominal heaviness, urinary retention, elevated CA-125 levels, and a 20 cm complex right-sided ovarian mass was diagnosed with mucinous carcinoma arising from a mature cystic teratoma. The case showed no ovarian surface involvement, nodularity, pseudomyxoma peritonei, or lymphovascular invasion. Immunohistochemistry showed positive results for CK20 and CDX2 but negative for CK7. Clinical and radiological workup did not reveal any neoplastic growth in the large intestine or appendix. The case of primary ovarian mucinous carcinoma originating from the gastrointestinal epithelium of a mature teratoma was diagnosed, and the patient was placed under follow-up care. Primary ovarian mucinous carcinoma originating from a teratoma, while infrequent, has been documented in the literature [19].

A 39-year-old woman with heavy menstrual bleeding and back pain presented with a 7 cm uterine tumor with bilateral 1.5 cm metastatic nodules in both ovaries, consistent with endometrial stromal sarcoma as shown in Figures 10A to 10D. The CD10 IHC marker showed diffuse positivity in the metastatic tumor deposit. Literature shows that the rate of ovarian metastasis of endometrial stromal sarcoma can be as high as 13%, as reported by Dos Santos LA et al. [20]. An uncommon case of small cell carcinoma of the ovary was seen in a 26-year-old woman with abdominal pain and ascites. She presented at an advanced stage (FIGO IIIc) and carries a dismal prognosis [21]. In our study, we found that benign tumors were more common in young females, while malignancies were more common in older individuals, as shown in Table 7. Although the age range of the patients with ovarian neoplasms varies, the frequency of malignancy increases as age progresses. This is corroborated by studies conducted by Jha R et al., Narang S et al., and Chanu SM et al. [22, 23, 24].

The study's retrospective design presents several limitations that impact the reliability of its findings. Firstly, the reliance on pre-existing clinical data may lead to missing information, particularly regarding symptom duration and family history of malignancies. Furthermore, the absence of a comprehensive radiological correlation limits the accuracy of diagnoses. Thirdly, the absence of follow-up data for ovarian malignancies restricts our ability to assess long-term effects and survival rates, which are critical for understanding the disease's progression.

## Conclusions

The clinicopathological analysis of ovarian neoplasms highlights the diverse nature of these tumors and their significant impact on women's health. Patients with ovarian neoplasms frequently present with abdominal pain. The demographic profile indicates that a majority of cases occur in women of reproductive age groups, with benign tumors like serous cystadenomas and mature cystic teratomas being the most frequently diagnosed. Serous carcinomas are more common in older postmenopausal women, usually presenting at an advanced stage (FIGO stage III or IV), and often carry a dismal prognosis. Diagnosing ovarian tumors of epithelial origin, especially those of serous and mucinous types, sometimes presents challenges. Extensive tissue sampling is required in difficult cases, particularly from solid-looking areas and mural nodules. Immunohistochemistry (IHC) markers such as PAX-8, P53, WT-1, ER, HNF-1beta, and napsin can also help resolve diagnostic dilemmas, along with molecular studies when required. Establishing accurate histopathologic diagnoses through these combined investigative approaches is critical for improving categorization. Further research is needed to refine diagnostic criteria and treatment strategies. A comprehensive understanding of ovarian neoplasms will enhance early detection efforts and lead to better survival rates for affected women.
